# An Assessment of Different Genomic Approaches for Inferring Phylogeny of *Listeria monocytogenes*

**DOI:** 10.3389/fmicb.2017.02351

**Published:** 2017-11-29

**Authors:** Clémentine Henri, Pimlapas Leekitcharoenphon, Heather A. Carleton, Nicolas Radomski, Rolf S. Kaas, Jean-François Mariet, Arnaud Felten, Frank M. Aarestrup, Peter Gerner Smidt, Sophie Roussel, Laurent Guillier, Michel-Yves Mistou, René S. Hendriksen

**Affiliations:** ^1^Agence Nationale de Sécurité Sanitaire de l'Alimentation, Maisons-Alfort Laboratory for Food Safety, University Paris-Est, Maisons-Alfort, France; ^2^European Union Reference Laboratory for Antimicrobial Resistance, National Food Institute, WHO Collaborating Center for Antimicrobial Resistance in Food Borne Pathogens and Genomics, Technical University of Denmark, Kongens Lyngby, Denmark; ^3^National Center for Emerging and Zoonotic Infectious Diseases, Centers for Disease Control and Prevention, Atlanta, GA, United States

**Keywords:** *Listeria monocytogenes*, WGS, cgMLST, wgMLST, SNPs, PFGE, conventional MLST, surveillance

## Abstract

**Background/objectives:** Whole genome sequencing (WGS) has proven to be a powerful subtyping tool for foodborne pathogenic bacteria like *L. monocytogenes*. The interests of genome-scale analysis for national surveillance, outbreak detection or source tracking has been largely documented. The genomic data however can be exploited with many different bioinformatics methods like single nucleotide polymorphism (SNP), core-genome multi locus sequence typing (cgMLST), whole-genome multi locus sequence typing (wgMLST) or multi locus predicted protein sequence typing (MLPPST) on either core-genome (cgMLPPST) or pan-genome (wgMLPPST). Currently, there are little comparisons studies of these different analytical approaches. Our objective was to assess and compare different genomic methods that can be implemented in order to cluster isolates of *L. monocytogenes*.

**Methods:** The clustering methods were evaluated on a collection of 207 *L. monocytogenes* genomes of food origin representative of the genetic diversity of the Anses collection. The trees were then compared using robust statistical analyses.

**Results:** The backward comparability between conventional typing methods and genomic methods revealed a near-perfect concordance. The importance of selecting a proper reference when calling SNPs was highlighted, although distances between strains remained identical. The analysis also revealed that the topology of the phylogenetic trees between wgMLST and cgMLST were remarkably similar. The comparison between SNP and cgMLST or SNP and wgMLST approaches showed that the topologies of phylogenic trees were statistically similar with an almost equivalent clustering.

**Conclusion:** Our study revealed high concordance between wgMLST, cgMLST, and SNP approaches which are all suitable for typing of *L. monocytogenes*. The comparable clustering is an important observation considering that the two approaches have been variously implemented among reference laboratories.

## Introduction

*Listeria monocytogenes* (*L. monocytogenes*) is one out of 17 species belonging to the genus *Listeria*, a Gram-positive rod-shaped bacterium (Weller et al., 2015). *L. monocytogenes* is classified into four major evolutionary lineages, 13 agglutination serotypes, and five molecular serotypes (Doumith et al., 2004; Orsi et al., 2011). *L. monocytogenes* is responsible for the serious foodborne illness, listeriosis caused by consumption of contaminated food such as unpasteurized milk, cheese, smoked salmon, uncooked meat and ready-to-eat food (Law et al., 2015). *L. monocytogenes* has the ability to grow at low temperatures, form bio-films and persist in food processing plants (Carpentier and Cerf, 2011). Subsequently, it represents a significant challenge for the food-producing industry (Ferreira et al., 2014). *L. monocytogenes* is one of the foodborne pathogens that cause the highest rate of mortality, yet its incidence is low (EFSA, 2014). Between 2008 and 2013, a significant increase of 8.6% in the incidence of listeriosis has been recorded in Europe. In 2015, over than 2200 cases were reported in Europe. This highlights *L. monocytogenes* as a serious re-emerging public health concern and it is therefore intensively monitored in developed countries (de Noordhout et al., 2014; EFSA, 2014).

The European surveillance system of *L. monocytogenes* from humans, foods, animals, and environments is still widely based on pulsed field gel electrophoresis (PFGE) (EFSA, 2014). PFGE was developed in the 1980s and the current PFGE scheme requires restriction by two enzymes using a validated standard protocol (Brosch et al., 1994; Michelon et al., 2015). PFGE has been extremely useful in *Listeria* outbreak investigations but its discriminatory power can be suboptimal for source tracking and source attribution (Ribot et al., 2006). The conventional multilocus sequence typing (MLST), based on the nucleotide sequence of seven house-keeping genes, provides a sequence type (ST) allowing strains to be clustered into clonal complexes (CC) (Ragon et al., 2008). Conventional MLST has been used in population diversity studies to investigate the population structure of *L. monocytogenes* (Chenal-Francisque et al., 2011; Haase et al., 2011; Cantinelli et al., 2013; Henri et al., 2016; Maury et al., 2016).

Recently, Whole genome sequencing (WGS)-based subtyping has proven to be extremely powerful for *L. monocytogenes*. A number of studies have demonstrated the advantages of using WGS analysis for national surveillance, outbreak detection and source tracking of *L. monocytogenes* (Chen et al., 2016a; Jackson et al., 2016). Single Nucleotide Polymorphism (SNP) and gene-by-gene approaches (genomic MLST) have been mainly employed at the genome scale. The gene-by-gene approach is based on inference of categorical data based on allelic variation of a predefined set of genes from either core genome only (called hereafter core genome MLST or cgMLST) or on a set of genes from both core and accessory genome (called hereafter whole genome MLST or wgMLST). The core genome consists of all genes present in all genomes of *L. monocytogenes* while the pan-genome consists of all the genes present in any strain of the species (supra-genome). Different cg or wgMLST schemes have been developed: in Germany (Ruppitsch et al., 2015), Austria (Hyden et al., 2016), and USA (Chen et al., 2016b), as well as by a consortium comprising the CDC (USA), the Pasteur Institute (France), the SSI (Denmark), PHAC Canada and PHE (UK) (Moura et al., 2016). The SNP approach is based on mapping raw sequence reads against a reference genome to call variations in both genes and intergenic regions. The choice of the reference genome is fundamental for SNP calling (Pightling et al., 2014). The SNP approach is currently used in Denmark (Agasan et al., 2013; Wingstrand et al., 2015; Jensen et al., 2016) and UK (Awofisayo-Okuyelu et al., 2016), as well as for regulatory purposes by the US Food & Drug Administration (FDA). An additional approach consists in inference of categorical data based on presence or absence of predicted proteins. Similar to the MLST approaches, the profile of presence and absence of predicted proteins could either be performed with the core genome (called hereafter cgMLPPST) or the pan genome (called hereafter wgMLPPST) (Leekitcharoenphon et al., 2014). Phylogenetic inference based on predicted proteins could be tested in order to cluster strains according to predicted phenotypic trait and adaptation abilities, and would be an original surveillance tool for source tracking (Deng et al., 2010).

The rapid implementation of WGS by different laboratories and laboratory networks using different approaches to analyse their data makes necessary to assess the differences between clustering methods. The main aim of this study was to assess the concordance between cgMLST, wgMLST, SNP, cgMLPPST, and wgMLPPST approaches using a well-defined panel of food strains of *L. monocytogenes* isolated in France during the last 20 years.

## Materials and methods

### Strain panel

Previously, we have investigated by PFGE and conventional MLST the genetic diversity of approximately 2000 *L. monocytogenes* of food origin isolated in France during the past 20 years. A panel of 207 *L. monocytogenes* strains from this study was selected to be statistically representative of the diversity of *L. monocytogenes*. It included strains isolated between 1989 and 2013, from various food matrixes and food processing environments. Out of the 207 strains, 127 isolates belonged to molecular serotype IIa, 25 to molecular serotype IIc, 17 to molecular serotype IIb, and 38 to molecular serotype IVb (Supplementary Table 1). The 207 *L. monocytogenes* strains belonged to 46 different STs and 38 distinct CCs (Supplementary Figure 1). The 207 strains represented 50 PFGE pulsotype clusters as depicted in the Supplementary Figure 2. The clusters were defined by the *Apa*1/*Asc*1 pulsotype patterns and clustered based on 80% similarity [unweighted pair-group method with arithmetic mean (UPGMA), with Dice's coefficient, tolerance and optimization set up at 1%; Henri et al., 2016]. The two reference strains, EGDe (accession number: NC_003210, ST35, CC9, serotype 1/2a and molecular serotype IIa) and EGD (accession number: HG421741, ST12, CC7, serotype 1/2a, and molecular serotype IIa) were included in the final set and used as reference for SNP calling. The complete list of the 209 genomes is available in Supplementary Table 1.

### DNA extraction and sequencing

DNA extraction was performed using Easy-DNA™ gDNA Purification Kit from Invitrogen™ (Life Technologies™ Headquarters, 5791 Van Allen Way, Carlsbad, CA 92008 USA). The DNA concentrations were measured using the Qbit dsDNA BR Assay Kit from Invitrogen™.

Libraries preparation and DNA sequencing were performed at the Welcome Trust Center for Human Genetics (Roosevelt Drive, Oxford OX3 7BN, 173 United Kingdom). Libraries were prepared by using the NEB library prep kits with in-house developed modifications. A sample of pooled libraries was loaded into Illumina HiSeq reagent cartridge with a standard flow cell. The 207 strains were subjected to pair-end sequencing. Insertion size of pair-end sequences ranged from 65 to 473 bp, with an average of 231 bp. The reads coverage ranged from 28 × to 442 ×, with an average of 213 × (Supplementary Table 1).

A biosample project was created as repository to store all raw sequence reads of this study with open access. The raw sequence data have been submitted to the European Nucleotide Archive (http://www.ebi.ac.uk/ena) under study accession no: PRJ 948.

### Genomic MLST

The wgMLST and cgMLST were performed at the Centers for Disease Control and Prevention, the USA (US-CDC) by the Enteric Diseases Laboratory Branch. The wgMLST scheme was developed from a set of over 200 annotated closed and high-quality draft genomes that represented the diversity of serotypes and lineages in *L. monocytogenes*. A total of 4,804 unique loci were identified to compose the wgMLST scheme, whereas 1,748 loci represent the cgMLST scheme. The cgMLST scheme was developed by the Pasteur Institute (Moura et al., 2016) and is available at PubMLST website (https://pubmlst.org/databases.shtml). The wgMLST with the cgMLST schema is included in the commercial software [BioNumerics v7.5 (Applied Maths NV, Belgium)]. Alleles were called for both the wgMLST and cgMLST schemes using BioNumerics v7.5. Unless raw reads (fastq format) were available, assembly-based allele calling (fasta format) was completed. The contigs were assembled using SPAdes 3.5.0, plug-in of the BioNumerics software v7.5. Alleles were named if genes fulfill the following criteria: a start and stop codon were present, the DNA sequence met the 85% minimum homology cut-off, there were no ambiguous base calls in the allele sequence, and had less than 100 gaps in the sequence alignment. Dendrograms of wgMLST and cgMLST were created using the UPGMA algorithm with the allele calls considered categorical data.

### Phylogenetic tree based on SNPs

The SNP tree was built with the pipeline CSI phylogeny accessible from the Center for Genomic Epidemiology (www.genomicepidemiology.org) (Leekitcharoenphon et al., 2012a; Kaas et al., 2014). The reference strains, EGD (ST35) and EGD-e (ST12) have been previously subjected to thorough genomic investigation and their differences are well documented (Bécavin et al., 2014). Both reference genomes belong to the same lineage II and serovar 1/2a but with different STs (ST35 and ST12, respectively).

The paired-end reads were mapped to the reference genomes using Burrows–Wheeler Aligner (BWA) (Li and Durbin, 2009). Initially, a SNP analysis was performed using the reference genome: EGD-e (accession number NC_003210, length 2,944,528 bp). Subsequently, a second SNP analysis was performed using the second reference genome: EGD (accession number HG421741, length 2,907,193 bp).

SNPs were determined using mpileup commands from SAMTools version 0.1.18. The SNPs were filtered according to five parameters: (1) a minimum distance of 10 bps between each SNP, (2) a minimum of 10x depth and 10% of the breadth coverage, (3) the mapping quality was above 30, (4) the SNP quality was higher than 20, and (5) all indels were excluded. For each genome, SNPs were concatenated to a single alignment corresponding to the positions of the reference genome.

The concatenated SNPs (with either EGD or EGD-e as reference) were inferred with the multi-core architecture (Aberer et al., 2010) of RAxML 8.2.4 (Stamatakis, 2014) based on a bootstrap analysis and search for best-scoring Maximum Likelihood tree with General Time-Reversible model of substitution and secondary structure 16-state model (Pattengale et al., 2009).

### Core and pan-genome plot

The raw reads were assembled using Velvet for *de novo* short reads assembly (Zerbino and Birney, 2008). Prediction of Open Reading Frames (ORFs) and proteins was performed using Prodigal in each *de novo* assembly (Hyatt et al., 2010; Jacobsen et al., 2011). Protein families were constructed by first aligning predicted proteins all-against-all using BLASTP with 50/50 rule (two genes were determined as a set if: the alignment length exceeds 50% of the longest sequence with more than 50% of the aligned sequences reported as identical) (Tettelin et al., 2005; Leekitcharoenphon et al., 2012b). Nonetheless, by this process, predicted proteins can be present in different families. Thus, all families sharing predicted proteins(s) were combined to ensure that each predicted proteins belongs to only one protein family (Tettelin et al., 2005; Friis et al., 2010; Lukjancenko et al., 2010, 2012; Vesth et al., 2010; Jacobsen et al., 2011; Kaas et al., 2012).

To each genome corresponds a set of predicted proteins, some of which are also found in other genomes. The pan-genome is the union of the predicted proteins, while the core genome is the intersection of the predicted proteins for the genomes under consideration (Tettelin et al., 2005; Leekitcharoenphon et al., 2012b). The size of the core- and pan genomes according to the number of genomes analyzed in our dataset is shown in Supplementary Figure 3.

### CgMLPPST tree

Multiple alignment for each core predicted proteins (predicted proteins found in all genomes) was performed with MUSCLE version 3.8.31 (Edgar, 2004). The concatenated aligned ORFs, without deletion of invariable positions, were obtained to reconstruct phylogenetic inference with the multi-core architecture (Aberer et al., 2010) of RAxML 8.2.4 (Stamatakis, 2014) based on a bootstrap analysis and search for best-scoring. Maximum Likelihood tree, with General Time-Reversible model of substitution and secondary structure 16-state model, was built (Pattengale et al., 2009).

### WgMLPPST trees

BlastP, using 50 percent length and 50 percent similarity rules, was performed for each samples against pan-genomes previously defined (Altschul et al., 1990). A profile of absence (0) or presence (1) of all genes was performed for each sample. The wgMLPPST tree was reconstructed from this matrix consisting of gene families (rows) and genomes (columns).

The analysis of presence/absence of the accessory genes across the 207 isolates showed that the genes could be divided into shell (genes that are frequently found) and cloud genes (genes that are rarely found). The wgMLPPST could be constructed by adding more weight either to cloud or shell genes. The trees were constructed using hierarchical clustering of the relative Manhattan distance according to the distance matrix (Snipen and Ussery, 2010; Leekitcharoenphon et al., 2012b).

### Trees visualization and annotation

All trees were visualized and annotated using iTOL (Letunic and Bork, 2007) and the R software (R Development Core Team, 2008). For better visualization, the trees were all circulated and the results of the standard typing approaches for each strain were displayed in outer external rings.

### Concordance between standard and genomic approaches

When all trees were reconstructed (the phylogenetic SNP, cgMLST and wgMLST, pan-genome, and core gene trees) we assessed the concordance of the genomic clustering with conventional groups: lineages, molecular serotype, PFGE pulsotype and ST's. The results were reported in percentage of concordance.

### Trees comparison and statistical analyses

A phylogenic tree can be characterized with two properties: the topology and the branch lengths. The topology is the branching structure of the tree and it indicates patterns of relatedness among strains. The comparison of the tree topology and distance were performed using the R packages “ade4,” “ape,” “dendextend,” “phangorn,” and “phytools” (Paradis et al., 2004; Dray et al., 2007; Schliep, 2011; Revell, 2012; Galili, 2015). “ade4” package was used for the graphical representation functions, “ape” package was used to read, plot and manipulate phylogenetic trees, “phangorn” and “dendextend” were used to compute pairwise distance between pairs of strains from phylogenetic network and “phytool” was used to visualize and analyse comparative data from species using colors.

### Cophenetic and the cor_cophenetic

The cophenetic is the distance between two strains and the exact height of the dendrogram where the two branches that contain the two strains join into one single branch. The cophenetic correlation (hereafter termed: cor_cophenetic) calculates the correlation between the cophenetic distance matrices of the two trees. The cor_cophenetic value ranges between −1 (perfect negative correlation) and 1 (perfect positive correlation). A value close to 0 (nil) indicates the absence of correlation for the two trees. The cophenetic and cor_cophenetic functions of dendextend and phangorn package were used to evaluate the clustering (Sokal and James, 1962; Cardona et al., 2013).

### The fowlkes-mallows index

The dendextend package calculates the Fowlkes-Mallows (FM) index which assess the similarity between two clusters (Fowlkes and Mallows, 1983). The FM index values are comprised between 0 (nil) and 1. The closer it is to 1, the more the clusters are similar. We calculated the asymptotic values, E_FM (Expected_Flowlkes-Mallows) and V_FM (Variance_Flowlkes-Mallows), expected under the null hypothesis (H0) that assumes that the two trees have the same topology if one tree is a random shuffle of the strains of the other tree (for instance no correlation between the trees). If E_FM+1,65·V_FM^0.5^ is below the observed one we can reject H0 at α = 0.05.

## Results

### Comparaison of the clustering efficiency of core and whole genome genomic MLST

**Figure 1:**
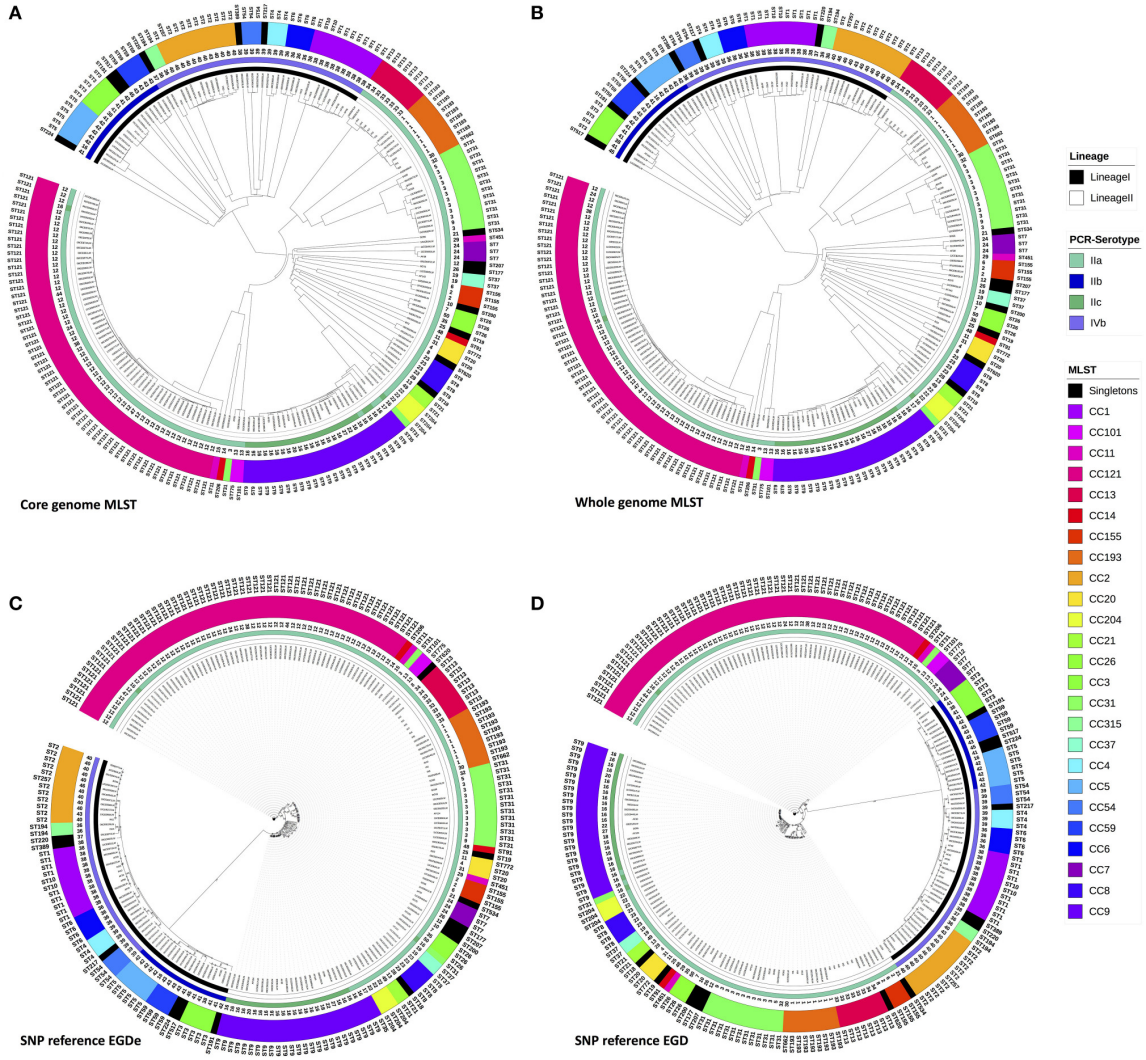
Phylogenic trees with the 208 *L. monocytogenes*, based on genomic MLST scheme define by Bionumerics® (core and pan) and Phylogenic trees based on SNPsvanalysis with EGD-e and EGD as references. Trees were circulated using ItoL. Inner circle represents lineage for each strains, second ring represents PCR-serotype, the third band shows the pulsotype cluster for each strain and the last two rings shows results from conventional seven loci MLST typing for each strains either with CC and ST. Color codes for Lineage, PCR-serotype and conventional seven loci MLST CC are shown aside in the figure legend. **(A)** The Analysis was performed on 1,748 core genes scheme from Bionumerics and dendograms was done using the UPGMA algorithm with the allele calls considered categorical data. **(B)** The Analysis was performed on pan genes scheme from Bionumerics and dendograms was done using the UPGMA algorithm with the allele calls considered categorical data. **(C)** SNP tree was constructed from SNPs that were identified using the pipeline CSI phylogeny accessible from the Center for Genomic Epidemiology (www.genomicepidemiology.org). EGDe was used as the reference genome to called SNPs. The SNP alignments were subjected to maximum-likelihood tree construction using PhyML with 100 bootstrap replicates. **(D)** SNP tree was constructed from SNPs that were identified using the pipeline CSI phylogeny accessible from the Center for Genomic Epidemiology (www.genomicepidemiology.org). EGD was used as the reference genome to called SNPs. The SNP alignments were subjected to maximum-likelihood tree construction using PhyML with 100 bootstrap replicates.

Initially, the cgMLST and wgMLST approaches were tested to infer the phylogeny of 207 food strains. Two major clades were observed for both cgMLST and wgMLST which corresponded mainly to lineage I and lineage II. Lineage II was subdivided in three clades that corresponded mainly to (1) CC13, CC193, CC31; (2) CC7, CC155, CC37, CC26, CC20, CC8, CC21, CC204, CC9, and seven singletons (ST19, ST18, ST177, ST200, ST207, ST534, ST620) (all singletons and CC from lineage II); and (3) to CC121 (lineage II) (Figures 1A,B). The inferred cgMLST and wgMLST phylogenies were in perfect accordance with the lineage classification whereas for the molecular serotyping, the concordance was slightly lower with a concordance of 96.6% for cgMLST and 97.6% for wgMLST (Table 1 and Supplementary Table 2). Importantly, the gene by gene approaches displayed a high concordance with conventional MLST i.e., 99.5% concordance with the cgMLST approach and 97.1% with the wgMLST (Table 1 and Supplementary Table 2). As expected the PFGE clustering showed a much lower performance with only 67.3 and 68.8% of concordance with cgMLST and wgMLST, respectively.

**Table 1:** Backward comparison with routine typing methods.

**Trees based on genomic methods**	**Lineage (%)**	**Serotype (%)**	**conventional MLST (%)**	**PFGE (%)**
Core genome MLST	100.0	96.6	99.5	67.3
Whole genome MLST	100.0	97.6	97.1	68.8
SNP tree EGD-e	100.0	99.0	94.7	69.2
SNP tree EGD	100.0	97.6	94.7	67.8
CgMLPPST tree based on the study panel	100.0	98.1	97.1	73.1
WgMLPPST tree (Shell)	99.0	96.6	87.50	62.5
WgMLPPST tree (Cloud)	83.2	88.0	87.02	70.2

*The performance of genomic methods was measured by concordance with routine methods (Lineage, PCR-Serotype, MLST, PFGE). The 100% means all strains from a particular group for routine method clustered together in corresponding tree. For instance, all strains clustered together according their lineage (I or II) for cgMLST, wgMLST, SNP trees and core genes tree but only 99 and 83.2% of strains for both MLPPST (respectively Shell and Cloud). See detail of count in Supplementary Table 2*.

**Figure 2:**
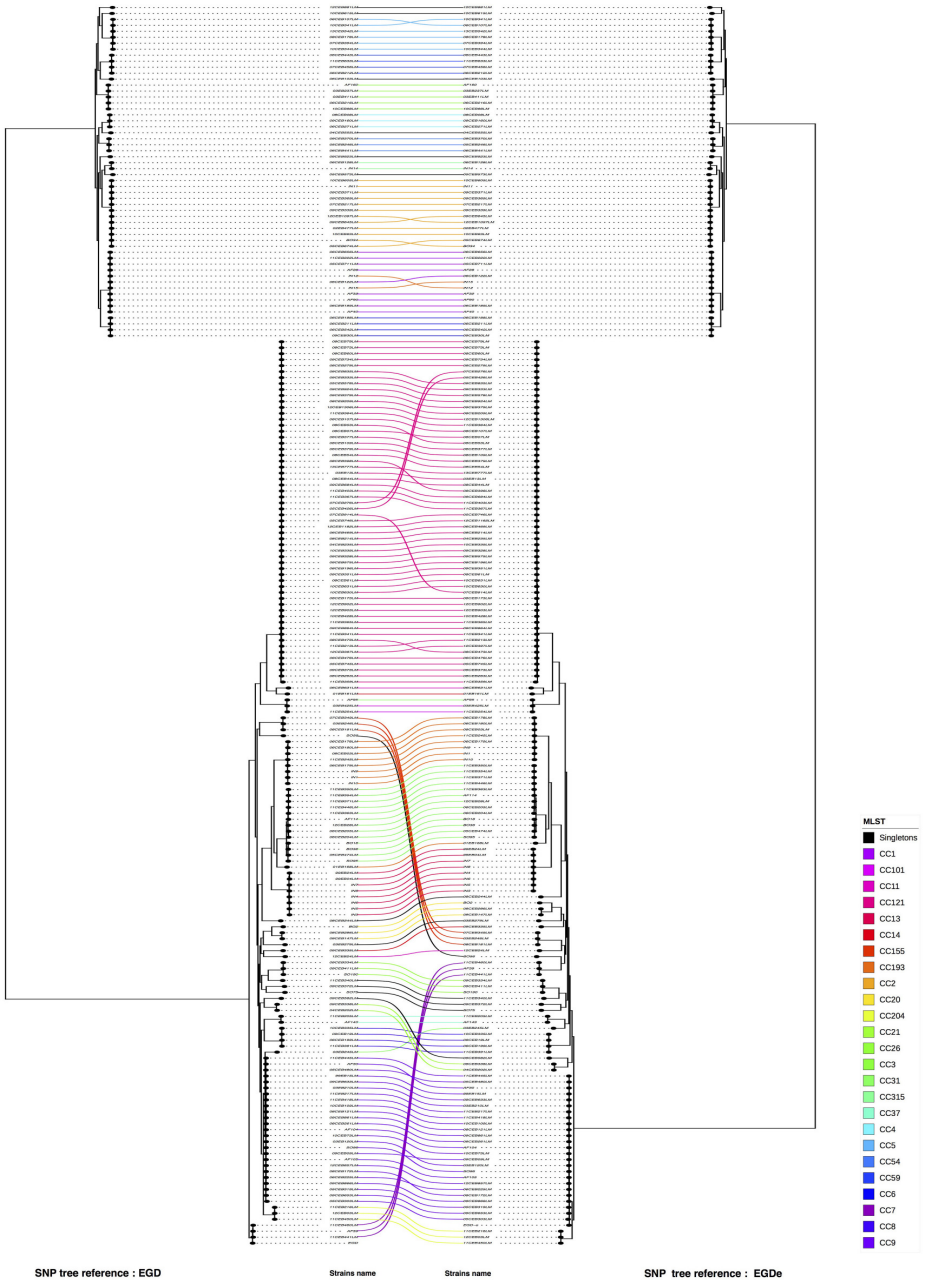
Visual comparison of genome SNP trees using EGD-e or EGD as reference. Using R software, SNP trees performed with the study panel of 208 *L. monocytogenes* were compared. By facing the two trees one in front of the other, corresponding strains were linked (on the left the SNP tree using EGD as reference and on right the SNP tree using EGD-e as reference). The connection between strains was colored according to the CC of the strains (refer to the color code). The two references are indicated in red. Nodes were rotated to optimize matching between corresponding strains in both trees as closely as possible. Similar clusters are connected by straight lines, while curved line connect strains from distinct clusters.

A visual comparison of cgMLST- and wgMLST-inferred phylogenies showed that strains from lineage II were grouped similarly and correctly with both approaches. To make the comparative analysis of the clustering methods easier, the trees to be compared were plotted facing each other with the same strains being connected (Figure 2). This data plot highlights the differences between phylogenies reconstructions. No clustering differences were observed in the shape of the trees (Figure 2), and only a few positioning differences were observed between strains within the same CC. The Fowlkes-Mallows Index and cor_cophenetic were calculated to quantify the similarity between the cgMLST and wgMLST inferred trees. In case of unrelated trees, the maximum expected value for FM index (E_FM) is 0.174 by taking into account E_FM and V_FM values. The calculated value of 0.885 is much higher than this critical value and indicates a high similarity between the two trees. In addition the calculated cor_cophenetic value of 0.999 (1 indicating a perfect correlation) statistically supports the conclusion that both methods lead to the same phylogenetic reconstruction.

### The SNP trees

The SNP trees were computed from concatenated SNPs identified from mapping raw reads to the reference genomes, EGD-e or EGD (Figures 1C,D). On average, 2.74 Mb (93.9%) of the EGD-e reference genome and 2.73 Mb (93.3%) of EGD reference genome were mapped against the 207 genomes included in the study. The phylogenies were inferred based on the analysis of 38,787 and 38,620 SNPs, using the EGD-e reference and the EGD reference, respectively. The SNP approaches grouped strains into two main clusters that corresponded to lineage I and II with a perfect 100% concordance (Table 1, Supplementary Table 2). When molecular serotypes were concerned, the concordance was of 99.0 and 97.6%, for SNP tree based on the EGD-e and the EGD references, respectively (Table 1, Supplementary Table 2). The SNP approaches were able to categorized strains according to STs (conventional MLST) with a concordance of 94.7% for both EGD-e and EGD references (Table 1, Supplementary Table 2). As expected, thePFGE clustering obtained the poorest concordance with the SNPs clustering with only 69.2 and 67.8%, for EGD-e and EGD references, respectively.

**Figure 3:**
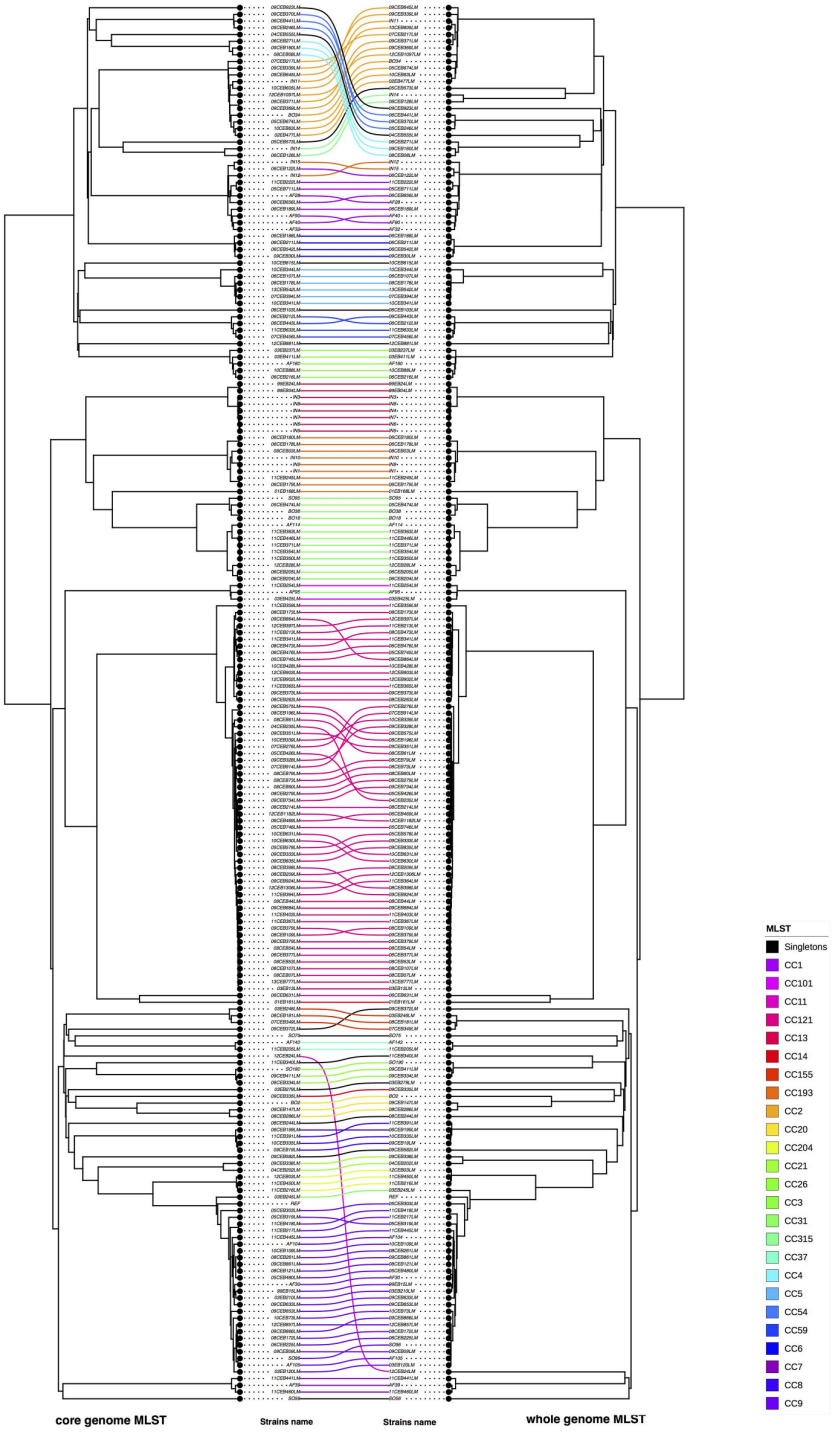
Visual comparison of cgMLST and wgMLST. R software was used to compare core genome and wgMLST on the study panel of 208 *L. monocytogenes*. In this opposite comparison corresponding strains were linked (on the left cgMLST and on right wgMLST). The connection between strains was colored according to the CC of the strains (refer to the color code). Nodes were rotated to optimize matching between corresponding strains in both trees as closely as possible. Similar clusters are connected by straight lines, while curved line connect strains from distinct clusters.

A visual comparison between the SNP analysis based on the EGD-e and EGD references showed that strains from lineage I are arranged in a similar way in the two trees, whereas strains from lineage II showed more variability. To assess the validity of these differences and remove artifacts, we performed a one to one plot with identical strains connected. To optimize matching, branches around nodes were also rotated (Figure 3). We noticed that only a few CC's (CC7, CC8 and CC155) and three unique strains (06CEB103LM, 09CEB923LM, and 11CEB445LM) changed positions in the two trees. The statistical analysis revealed that the two trees were similar as the FM index of 0.796 was higher than the E_FM value (0.409). Likewise, the cor_cophenetic equal to 0.999 confirmed the highly similar tree topologies. Finally, the analysis indicated that changing reference for SNP calling produce similar but not identical trees. By comparison, the FM index (0.885) for the wgMLST-cgMLST clustering comparison was 0 closer to 1.

### Comparison between the SNP and genomic MLST

**Figure 4:**
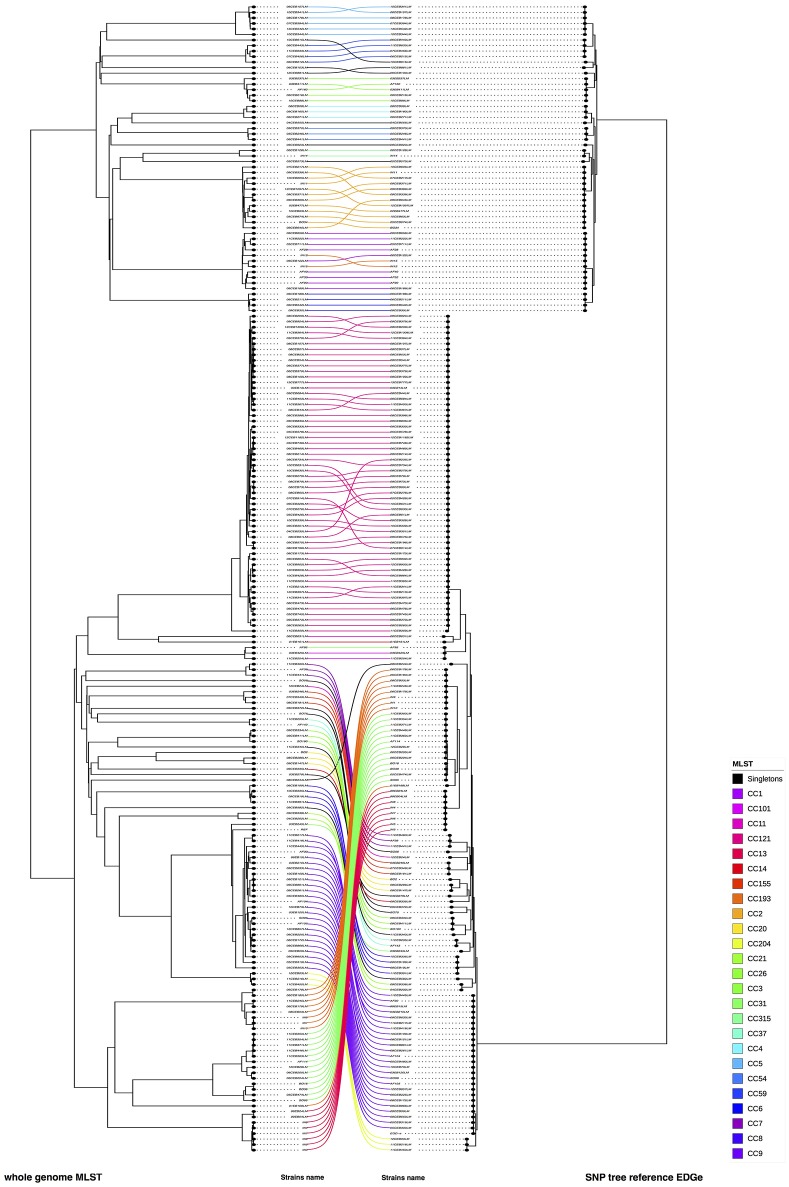
Visual comparison of genome SNP and wgMLST. We compared genome SNP and wgMLST on the study panel using R software (on the left cgMLST and on right wgMLST). Using this face-to-face comparison, we linked corresponding strains. The connection between strains was colored according to the CC of the strains (refer to the color code). Nodes were rotated to optimize matching between corresponding strains in both trees as closely as possible. Similar clusters are connected by straight lines, while curved line connect strains from distinct clusters.

We compared the phylogenic trees based on SNPs with cgMLST and wgMLST approaches, respectively. (Figure 4, Supplementary Figure 4). In both comparisons, we observed that six CC's (CC5, CC59, CC8 for lineage I and CC13, CC31, CC193 for lineage II) and three unique strains changed of position in the compared trees (08CEB244LM and IN12 for both comparisons, 10CEB615LM and 05CEB573LM for SNP vs. wgMLST and SNP vs. cgMLST, respectively). The FM Index (0.486) was low but higher than the expected E_FM (0.135) value, providing statistical evidence that the SNP and the wgMLST approaches provide overall similar results. The same conclusion was reached when the SNP and the cgMLST approaches were compared (FM Index of 0.426; with E_FM = 0.146). The cor_cophenetic was not estimated as the two matrices of distance are not based on the same distance scale.

### CgMLPPST tree

The cgMLPPST, as opposed to the allele-based cgMLST tree, was inferred based on the multiple alignment for each core genes found among the genomes included in the study (Supplementary Figure 5). This approach showed 100, 97.1, and 98.1% concordances with lineage, conventional MLST, and molecular serotype, respectively (Table 1 and Supplementary Table 2). Overall, the cgMLPPST performed better than these conventional methods (Table 1, Supplementary Table 2).

The core genes determined in this study might be seen as large due the number of genomes used (129 182 variable positions across 207 genomes). With more genomes, from diverse origin, and if our panel would include strains from lineage III and IV; the number of core genes would probably be lower than 2000 genes and should approach the 1748 genes used in the cgMLST scheme. (Supplementary Figure 3). Indeed, this result indicates that the panel of food strains do not represent the full diversity of *L. monocytogenes*.

### WgMLPPST approaches

We observed five major clades in the wgMLPPST trees (Supplementary Figures 6, 7). The five clades corresponded mainly to (1) lineage I, CC121, (2) CC193, CC31, (3) CC13, (4) CC9, CC204, and (5) CC7, CC8, CC20, CC21, CC21, CC26, CC31, CC37, CC101, and seven unique strains from lineage II (ST19, ST18, ST177, ST200, ST207, ST534, ST620). For the wgMLPPST approaches, we observed that the phylogenetic trees obtained from either the shell or the cloud failed to assign one and three strains to the correct lineage (Table 1, Supplementary Table 2). Additionally, strains from different molecular serotypes were interspersed causing a low concordance with molecular serotypes (Table 1, Supplementary Table 2). Furthermore, the concordance between conventional MLST and wgMLPPST approaches displayed the lowest scores among genomic methods (Table 1, Supplementary Table 2). Like for the previous genomic approaches, the concordance with PFGE clustering were low and decreased to 62.5 and 70.2%, for shell and cloud wgMLPPST, respectively. Those results indicate that wgMLPPST is not relevant for surveillance purpose as strains from different lineage, ST and molecular serotype can be mixed. WgMLPPST approaches failed to group together strains from the same lineage despite their genetic homogeneity (Orsi et al., 2011; Paul et al., 2014). The failure to cluster strains from the same lineage confirmed that wgMLPPST is not suitable for phylogeny and routine surveillance purposes (Leekitcharoenphon et al., 2014).

## Discussion

The speed, cost and efficiency of WGS make it a realistic alternative to most current phenotypic and molecular typing methods for surveillance and outbreak investigation of foodborne pathogens. Currently, WGS is being implemented as a routine diagnostic tool and for surveillance and outbreak detection purposes in a few countries around the world enhancing the public health preparedness. In Europe, EFSA has recognized the strength and power of WGS and already launched pilot projects targeting *L. monocytogenes* and expanding to other foodborne pathogens (Nielsen et al., 2017). There are however, some limitations and obstacles present for the immediate use of WGS for surveillance of foodborne pathogens purpose such as harmonization of the phylogenetic approached, assigning an appropriate nomenclature, and sharing data (EFSA, 2014). For this reason, many European and National projects are currently concentrating their efforts on developing WGS protocols and workflows. The objective of this study was to assess and compare genomic MLST, genomic SNP, predicted protein core- and pan-genomic approaches using a unique and diverse panel of *L. monocytogenes* strain including 36 clonal complexes isolated from food.

The backward comparison to PFGE, lineage and molecular serotype showed that all genomic approaches used in our study: cgMLST, wgMLST, and SNP analyses provide equally reliable results. Our assessment also included the analysis of discrepancies between cgMLST and wgMLST, as well as the influence of the chosen reference genome for SNP investigations. Hence, a strenuous question is the choice of applying SNP analysis vs. genome MLST.

The comparison between the two genome MLST methods, indicate highly similar phylogenetic tree reconstruction regarding both distance and clustering. However, the ease of use of the two methods is not the same. The cgMLST scheme contains a well-defined set of species-wide conserved genes. A precise and calibrated cgMLST is particularly stable, hence suits especially routine epidemiology. This stability may not be provided by wgMLST because of the pan-genome variability and potential continuous expansion. The pan-genome of *Listeria* was calculated several times and comprised between 3,056 genes and 7,000 genes, indicating that it will be necessary to reach a global consensus to define the accessory genes that are part of the whole genome MLST scheme (Deng et al., 2010; Maury et al., 2016). However, the development of methods combining the stability of the core scheme with the accessory genes could certainly be helpful in situation where it is necessary to increase discriminatory power beyond the cgMLST (Maiden et al., 2013).

In a study on a single strain and in a prospective surveillance study of *L. monocytogenes*, it was reported that the choice of the reference genome affects the results of SNP analysis (Pightling et al., 2014). We have extended the investigation to 207 genomes to measure the impact that this reference choice can have on phylogenetic tree reconstruction. Our results showed that the distances between two sets of strains are statistically identical whatever the chosen reference genome, however it impacts the positioning of small groups of strains (Figure 2) probably because of unstable transient variants which are retained is this analysis and/or intergenic variants which provide additional discrimination power. One solution to avoid these differences into the tree topologies, would be to remove transient variants from the SNP dataset. *L. monocytogenes* is a clonal species and conventional MLST has proved it robustness for population structure (Ragon et al., 2008; Maury et al., 2016). We believe that the use of SNP analysis for global epidemiological purpose would require a global consensus on a set of CC-specific genomes that could be used as references to perform SNP-calling within ST- or CC-groups. The use of multiple reference genomes would increase the discriminatory power of the method for each CC. Furthermore, using SNP-based phylogeny specific SNP markers, could be proposed to discriminate ST or CC. This SNP-based barcode could cover all main lineages, ST and could classify strains in sub type within ST (Coll et al., 2014). For greater accuracy and efficiency at an international level this should be accompany with the use of a common SNP calling pipeline (Bertels et al., 2014), determining if the variants induced by recombination events must be removed, or not, from the variant dataset before phylogenetic reconstruction (Hedge and Wilson, 2014).

Our results demonstrate with a strong statistical support that the SNP and genomic MLST approaches led to similar phylogenetic reconstruction. This provides microbiologists and epidemiologists working on cluster analysis of *L. monocytogenes* two alternative methods with almost the same discriminatory power and precision. Remarkably, most of the discrepancies observed in the topology concerned full CC or ST. This result shows the noticeable clonality of *L. monocytogenes* and also the robustness of the conventional MLST for population structure since strains of the same CC or ST cluster together irrespective of the genomic methodology used. This study did not find any difference in the discriminatory power of the SNP and the genomic MLST approaches. Despite that the two approaches give similar results, the SNP and genome MLST entail different advantages and disadvantages which should be taken into account in a global epidemiological perspective. None of the approaches require a substantial amount of time and substantial bioinformatics expertise, indeed wgMLST is commercially available from Bionumerics® (and cgMLST in public domain) and numerous open-source SNP calling pipelines are available.

The main difference between the two approaches is that a database of loci and associated alleles is used to identify alleles for cg/wgMLST whereas one reference strain is used for SNP calling. An important benefit of the classification of isolates with cg/wgMLST is that it would be stable over time as new isolates are added, on the other hand it requires a careful curation of new alleles. An additional significant advantage is that the cg/wgMLST can provide a genome sequence type which could lead to a common nomenclature, provided that timely update of alleles databases between servers are adopted. A common nomenclature and a stable scheme should ease data portability and sharing making communication more effective. Allelic database management requires extensive curation (Jolley et al., 2010) which for the most part can be automated with little manual interference. For these reasons, the genomic MLST approaches appear to be better suited for the use in laboratory surveillance of listeriosis where direct comparability of analytical results by different laboratories is critical, e.g. for global outbreak detection and investigation.

Concerning the SNP-based approaches, a higher discrimination would necessitate the use of different reference genomes for routine surveillance. However, the SNP approach can be fully automated while a question mark remains concerning the automation of the curation process of cg/wgMLST alleles database (Leekitcharoenphon et al., 2012a; Moura et al., 2016). Theoretically, SNP is also more discriminative by taking into account intergenic sequences but it is also more sensitive to parameters variations (reference, SNP calling filters, coverage) inducing divergence in topology of trees as shown in our study (Pightling et al., 2014). It must also be noticed that the SNP-based approaches give the opportunity to detect recombinaison evens (Croucher et al., 2015; Didelot and Wilson, 2015).

As discussed and highlighted in this work the topology of trees is made of branching and distances between strains. These two parameters provide a precise idea of the relationship between strains. This network is used to set-up groups of more and less related strains. Hence, another point of importance is to define thresholds to guide the identification of clusters of related isolates, in a way similar to what has been defined for ST or CC in MLST. This question should be addressed to implement routine surveillance (number of alleles variations for genomes MLST to define an ST or number of SNPs difference for SNP approaches) and a recent study has proposed some answers (Nielsen et al., 2017). An allelic difference threshold for genomic MLST for point source outbreaks has been proposed by Moura and colleagues (Moura et al., 2016) to defined cgMLST type (CT). However, although firm cluster definition criteria may be defined for contamination event point-source outbreaks, it is not possible to define universal cluster criteria for outbreaks that are caused by persistent contamination of a production environment because of the diversity of the situations that enables outbreak strains to evolve and diversify over time (Chen et al., 2017).

The difficulty to define SNP/allele threshold was recently highlighted by Chen et al. (2017) who investigate an outbreak linked to cheese in the USA. In this thorough study, the authors strongly advise to combine multiple WGS analyses (i.e., SNP and allele calling) with relevant phylogenetically reconstruction procedures to confidently delineate related and unrelated isolates (Chen et al., 2017).

Finally, the development of SOP (Standard operating procedure) for production and analysis of WGS data is of paramount importance in order to reach sound conclusions that will be confidently handled by the risk management authorities. In that perspective, the indexes we used in this study to compare clustering and topology will be valuable tools to set out SOP for WGS analysis in the field of microbiological food safety.

## Conclusion

The backwards comparability between the standard MLST methodology and the genomic MLST and SNP approaches were essentially perfect. Because genomic MLST or SNP approaches provide better resolution, WGS can replace PFGE as the new gold standard for epidemiological typing of *L. monocytogenes*. Moving into the genomic era, it is vital to keep a focus on enhancing the genomic technology, to produce “plug and play solutions” and to provide the technology to diagnostic laboratories responsible for outbreak detection and surveillance. Our results showed concordance between the phylogenetic clustering of *L. monocytogenes* by the genomic MLST and SNP approaches; they are statistically similar in term of tree topology and could be used in combination when facing complex epidemiological situations.

## Author contributions

CH was in charge of the whole project and participated in data production, data interpretation, and drafting the manuscript. PL contributed to the data production, data interpretation. HC contributed to the data production and drafting of the manuscript. NR participated to data production. RK participated to data production J-FM participated to the DNA extraction. AF participated to the genomic data production of SNP. FA participated to the design of the study and drafting the manuscript. SR participated in the design of the study. PG. participated in the drafting of the manuscript. LG participated to the study design and design the statistical analysis and contributed in drafting the manuscript. M-YM and RH participated in the design and coordination of the study and in drafting the manuscript. All authors read and approved the final manuscript.

### Conflict of interest statement

The authors declare that the research was conducted in the absence of any commercial or financial relationships that could be construed as a potential conflict of interest.
