# Supplementary figures and images for: An Assessment of Different Genomic Approaches for Inferring Phylogeny of Listeria monocytogenes
Source: Front Microbiol. 2017 Nov 29;8:2351. doi: 10.3389/fmicb.2017.02351 (PMC5712588; doi:10.3389/fmicb.2017.02351)

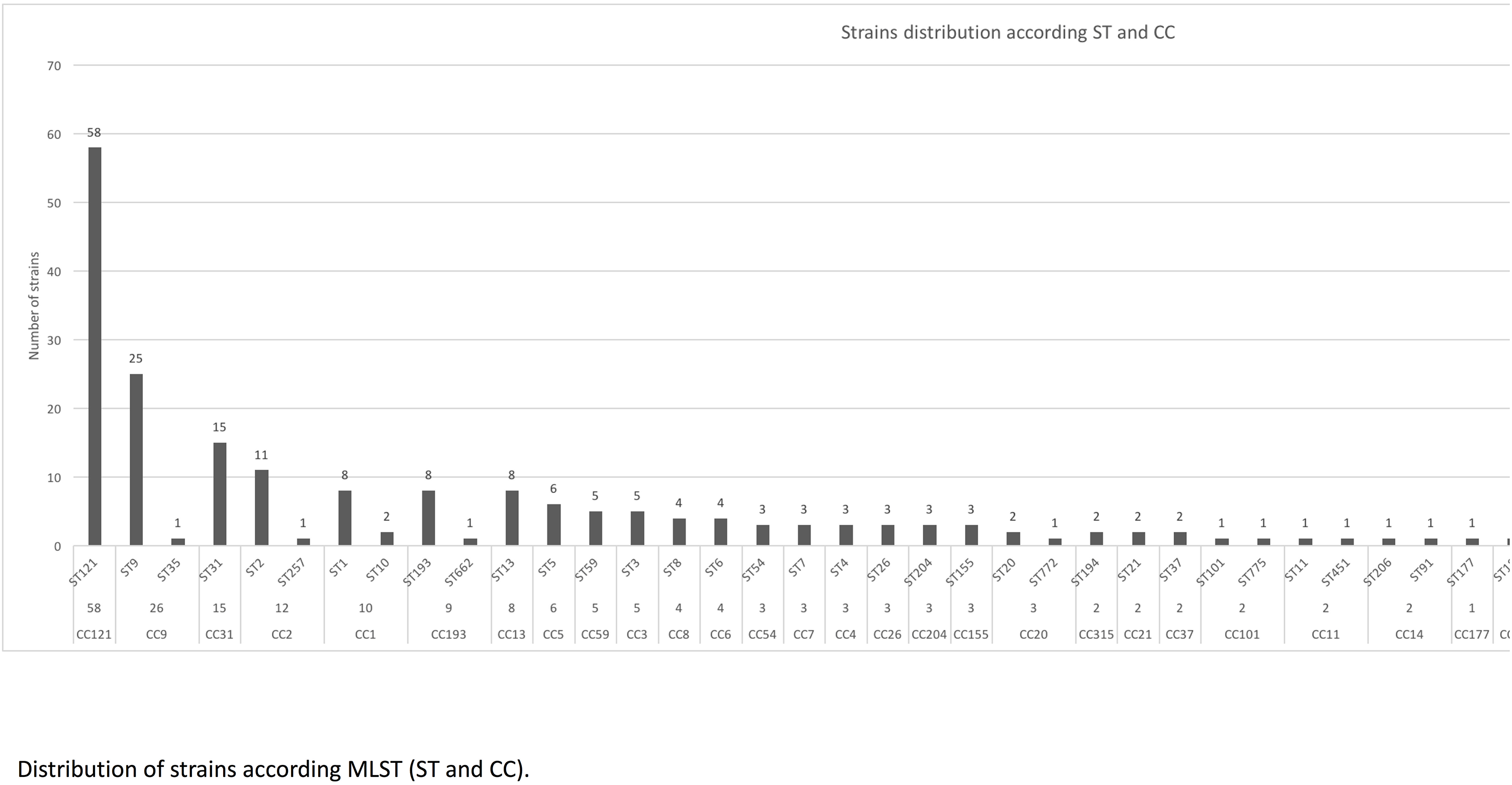

Supplement: Supplementary file 1 [file Image1.TIFF]

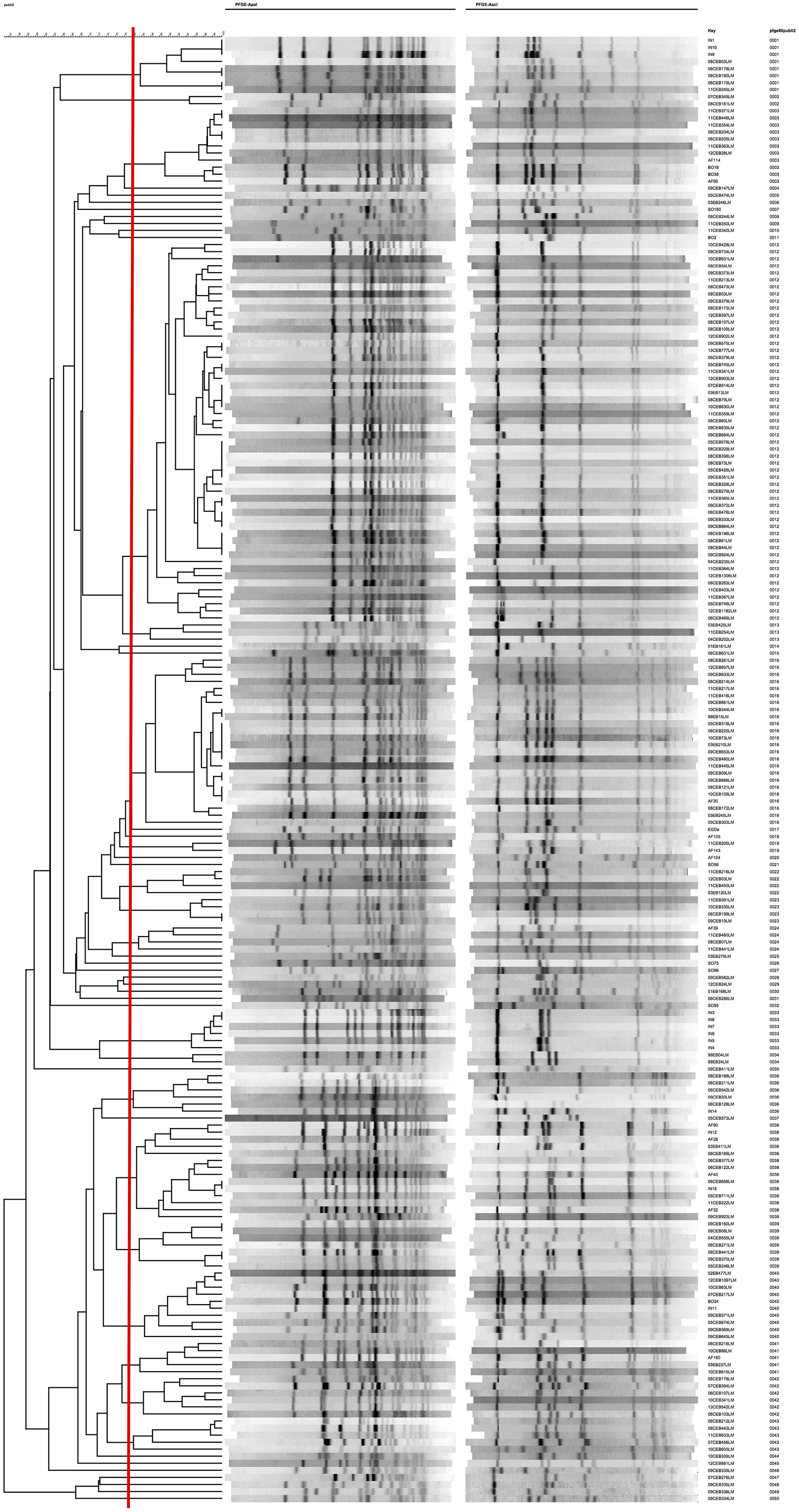

Supplement: Supplementary file 2 [file Image2.TIFF]

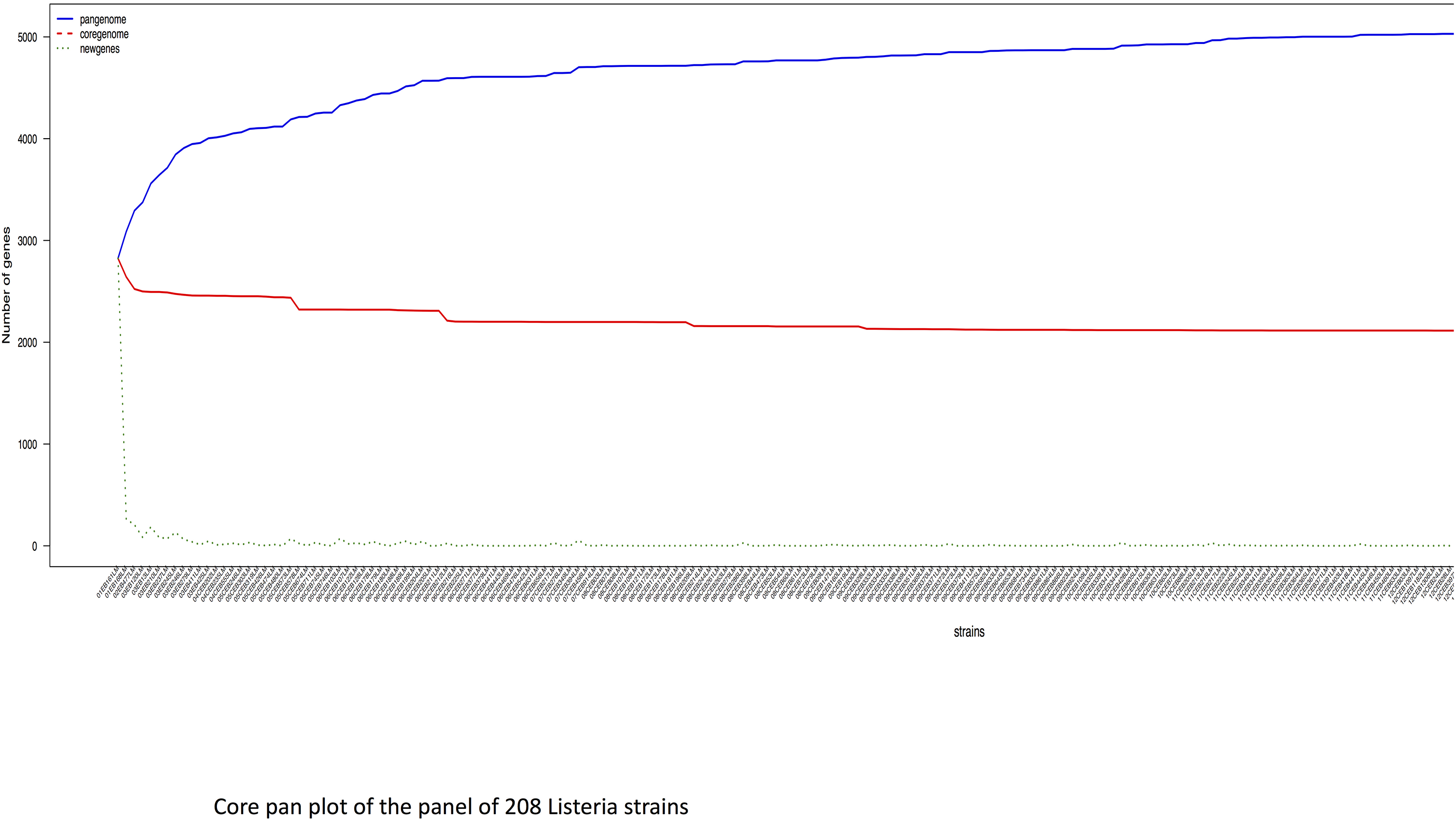

Supplement: Supplementary file 3 [file Image3.TIFF]

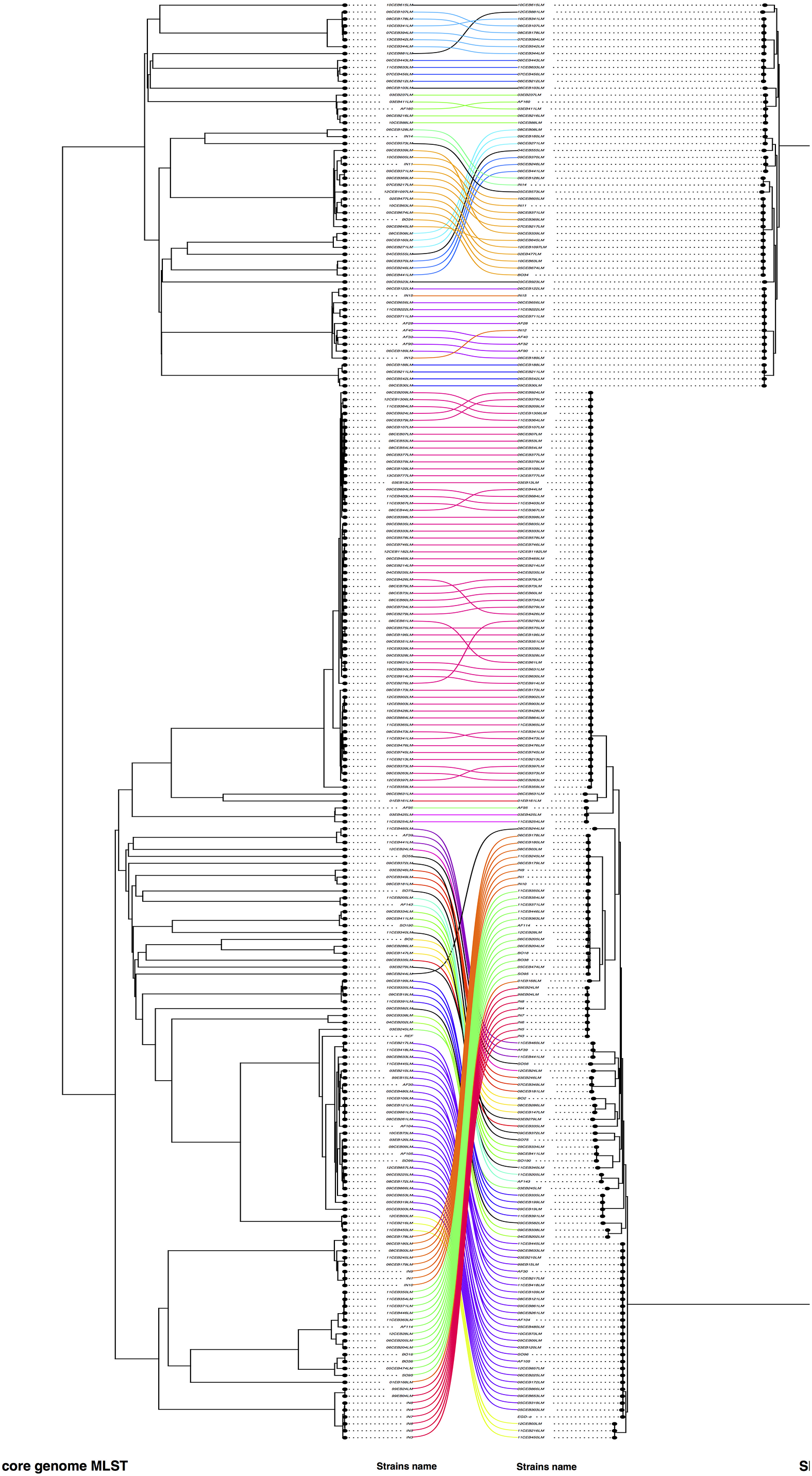

Supplement: Supplementary file 4 [file Image4.TIFF]

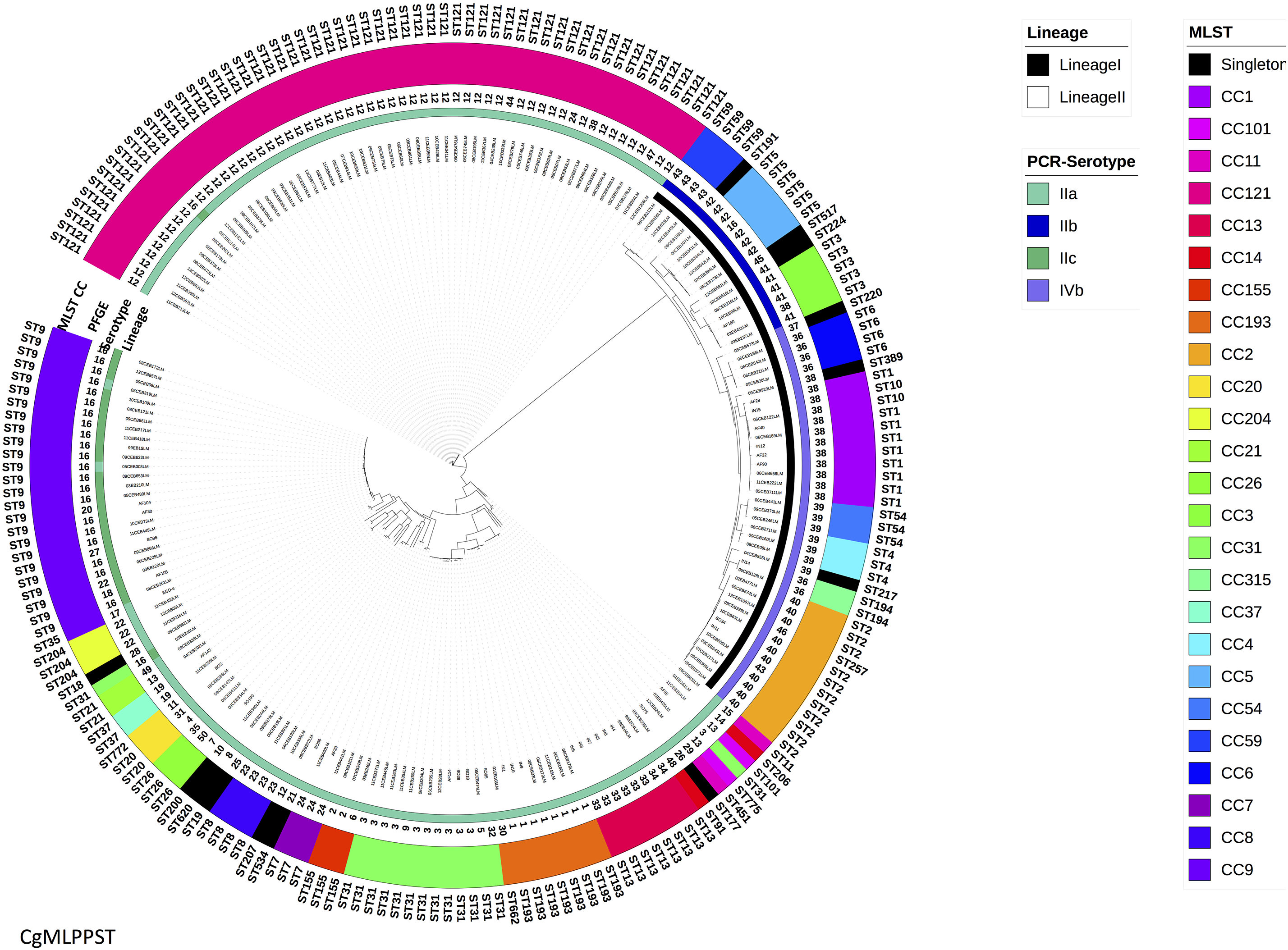

Supplement: Supplementary file 5 [file Image5.TIFF]

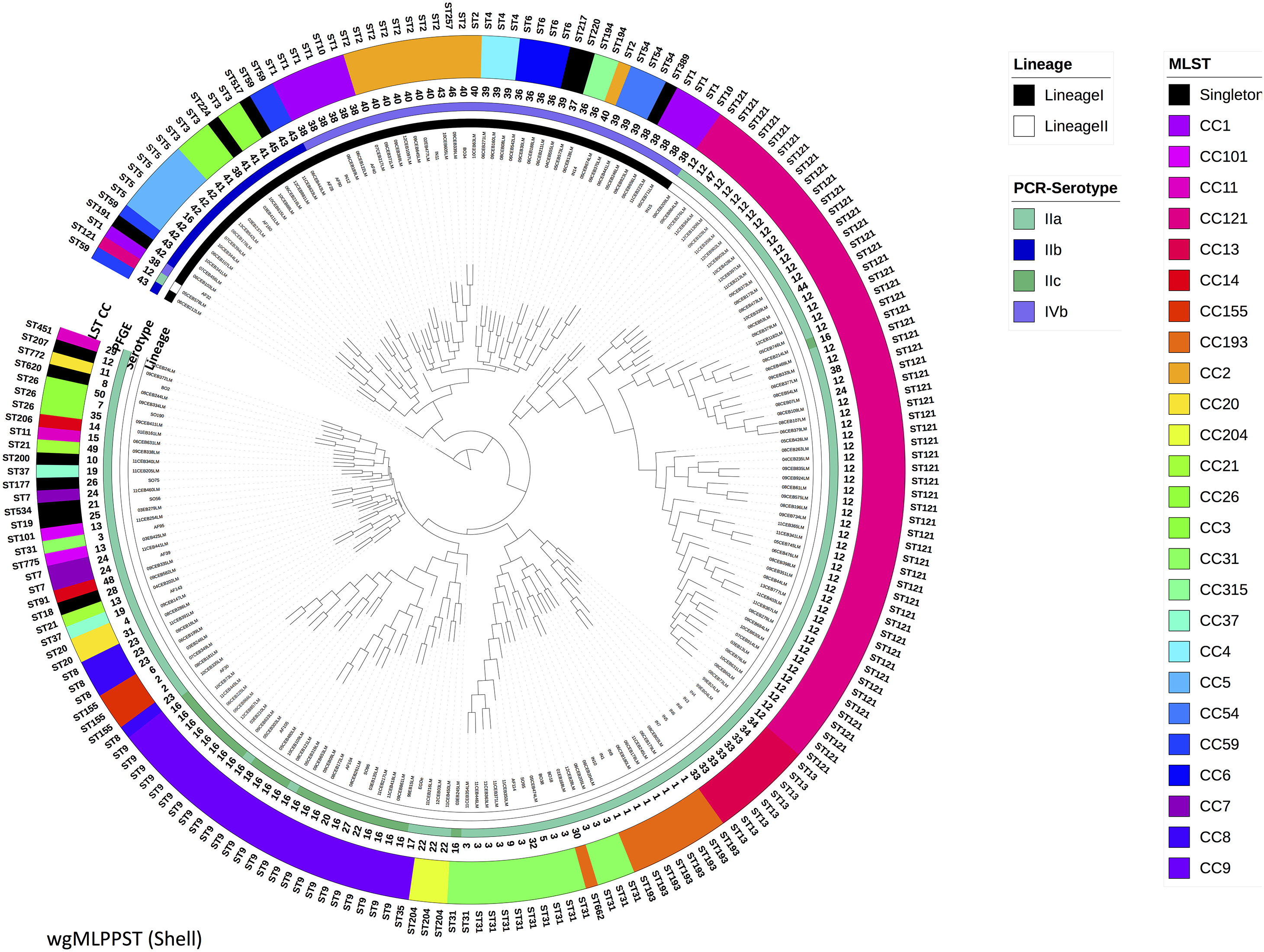

Supplement: Supplementary file 6 [file Image6.TIFF]

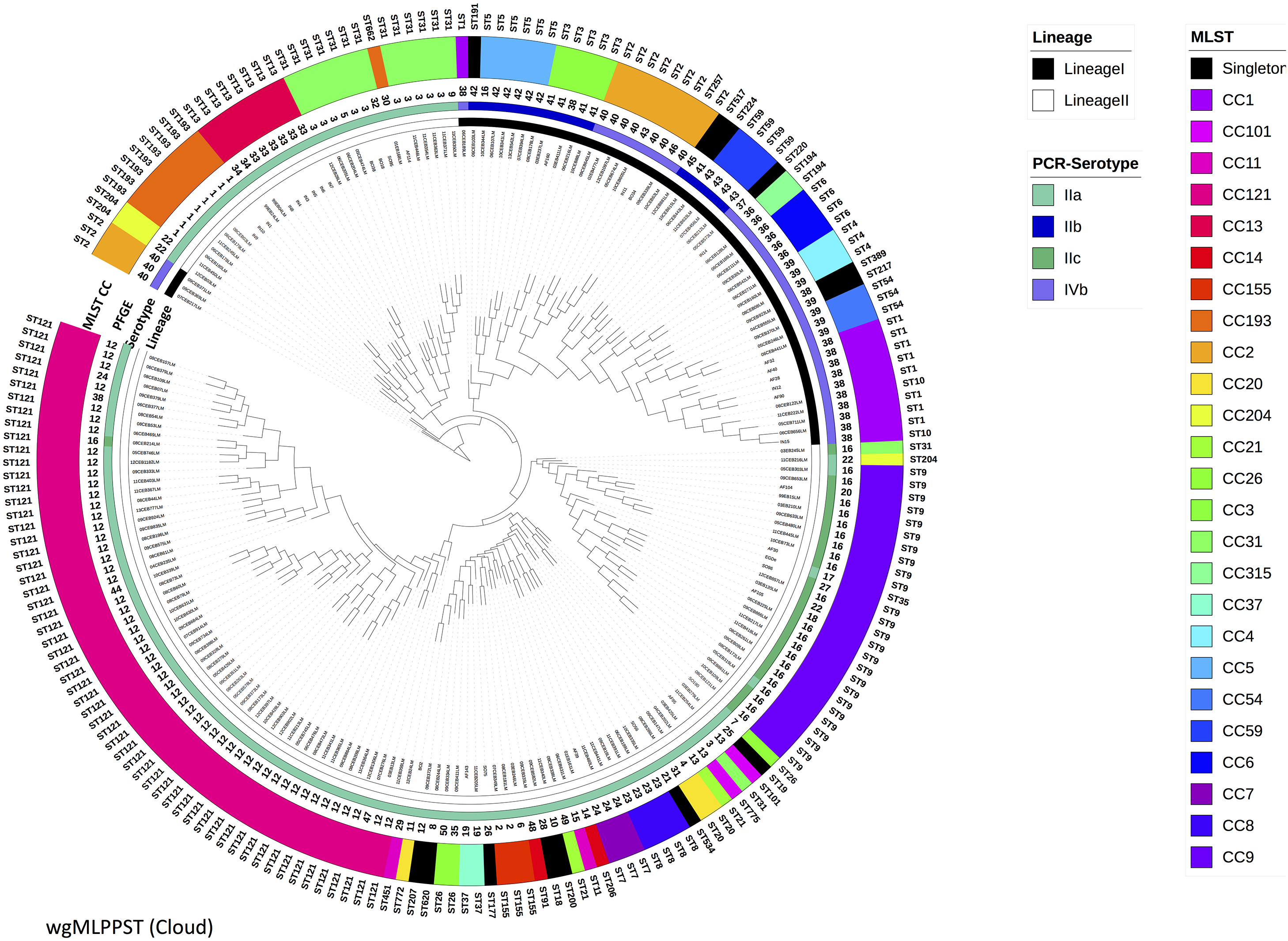

Supplement: Supplementary file 7 [file Image7.TIFF]
