# Supplementary material for: An Assessment of Different Genomic Approaches for Inferring Phylogeny of Listeria monocytogenes
Source: Front Microbiol. 2017 Nov 29;8:2351. doi: 10.3389/fmicb.2017.02351 (PMC5712588; doi:10.3389/fmicb.2017.02351)
Supplement: Supplementary file 10 [file Presentation1.PDF]

Supplementary Material

An Assessment of different Genomic Approaches for Inferring Phylogeny of Listeria monocytogenes

Clémentine Henri, Pimlapas Leekitcharoenphon, Heather A. Carleton, Nicolas Radomski, Rolf S. Kaas, Jean-François Mariet, Arnaud Felten, Frank M. Aarestrup, Peter Gerner Smidt, Sophie Roussel, Laurent Guillier, Michel-Yves Mistou1*, René S. Hendriksen

***: Corresponding author**: Michel-Yves Mistou

University Paris-Est, Anses, Maisons-Alfort Laboratory for Food Safety, Maisons-Alfort, France.

Phone: +45 35 88 60 00
Fax: +45 35 88 60 01

E-mail: michel-yves.mistou@anses.fr

1 Supplementary Data

No supplementary data

2 Supplementary Figures and Tables

2.1 Supplementary Figures

Supplementary Figure 1: Distribution of strains according conventional seven loci MLST (ST and CC). The number of strains per ST is shown on top of bar. STs included in the same CC were gather and cumulative strains numbers is indicated in the data table below the graph. CC were ranked by number of strains.

Supplementary Figure 2: Dendrogram of PFGE pulsotype at 80% similarity. All strains were typed and interpreted by PFGE using the protocol previously described in Félix et al.. For each of the strains, a combined PFGE pulsotype was defined based on the ApaI pulsotype/AscI pulsotype. The ApaI and AscI pulsotypes were considered different if there was at least one different band. Pulsotype clustering was composed of profiles with 80% similarity (unweighted pair group method with arithmetic average [UPGMA]), with Dice's coefficient, tolerance, and optimization set at 1%. The pulsotype clustering and the number of strains in each cluster were established according to PFGE dendrogram clustering using a BioNumerics® clustering script, and a numerical code was assigned to each cluster shown in the figure.

Supplementary Figure 3: Pan- and core-genome plot of the 208 *L. monocytogenes* from the study panel. Supplementary figure 3 shows an increase of the pan-genome (5118 gene families) and a slight decrease of the core-genome (2017 gene families) as more *L. monocytogenes* genomes were added from the study panel. Genomes were sorted according Lineage and ST.

Supplementary Figure 4: Visual comparison of genome SNP and cgMLST. We compared genome SNP and cgMLST on the study panel using R software (on the left cgMLST and on right wgMLST). Using this two by two comparison, we linked corresponding strains. The connection between strains was colored according to the CC of the strains (refer to the color code). Nodes were rotated to optimize matching between corresponding strains in both trees as closely as possible. Similar clusters are connected by straight lines, while curved line connect strains from distinct clusters.

Supplementary Figure 5: CgMLPPST tree based core gene defined with the study panel. CgMLPPST tree based core gene defined with the study panel was built with FastMe and condense using PHYLIP solfware. For graphical representation final condensed tree was circulated using ItoL. Inner circle represents lineage for each strains, second ring represents PCR-serotype, the third circle shows the pulsotype cluster for each strain and the last two rings shows conventional seven loci MLST typing results for each strains CC and ST. Color codes for Lineage, PCR-serotype and conventional seven loci MLST CC are shown aside.

Supplementary Figure 6: Phylogenic tree based on wgMLPPST (shell). The shell wgMLPPST tree was reconstructed from this matrix consisting absent/presence of pan-genome gene families and tree was constructed using hierarchical clustering of the relative Manhattan distance according the matrix. The Shell wgMLPPST tree was circulated using ItoL. Inner circle represents lineage for each strains, second ring represents PCR-serotype, the third circle shows the pulsotype cluster for each strain and the last two rings shows conventional seven loci MLST typing results for each strains CC and ST. Color codes for Lineage, PCR-serotype and conventional seven loci MLST CC are shown aside.

Supplementary Figure 7: Phylogenic tree based on wgMLPPST (cloud). The cloud wgMLPPST tree was reconstructed from this matrix consisting absent/presence of pan-genome gene families and tree was constructed using hierarchical clustering of the relative Manhattan distance according the matrix. The cloud wgMLPPST tree was circulated using ItoL. Inner circle represents lineage for each strains, second ring represents PCR-serotype, the third circle shows the pulsotype cluster for each strain and the last two rings shows conventional seven loci MLST typing results for each strains CC and ST. Color codes for Lineage, PCR-serotype and conventional seven loci MLST CC are shown aside.

2.2 Supplementary Tables

Supplementary Table 1: Details table of strains study panel with corresponding metadata.

Supplementary Table 2: Details tables of mismatches counting for backward compassion between routine typing methods and genomic methods.

Table 2a: shows visual discordances between lineage and cgMLST tree, wgMLST tree, SNPEGDe tree, SNPEGD tree, coregene tree, Cloud pan-genome tree and Shell pan-genome tree clustering.

Table 2b: shows visual discordances between PCR-serotype and cgMLST tree, wgMLST tree, SNPEGDe tree, SNPEGD tree, coregene tree, Cloud pan-genome tree and Shell pan-genome tree clustering.

Table 2c: shows visual discordances between MLST and cgMLST tree, wgMLST tree, SNPEGDe tree, SNPEGD tree, coregene tree, Cloud pan-genome tree and Shell pan-genome tree clustering.

Table 2d: shows visual discordances between PFGE pulsotype clustering at 80% similarity and cgMLST tree, wgMLST tree, SNPEGDe tree, SNPEGD tree, coregene tree, Cloud pan-genome tree and Shell pan-genome tree clustering.

Table 2e: summaries visual discordances between lineage, PCR-serotype, MLST, PFGE pulsotype clustering at 80% similarity and cgMLST tree, wgMLST tree, SNPEGDe tree, SNPEGD tree, coregene tree, Cloud pan-genome tree and Shell pan-genome tree clustering first in absolute value, then in percentage of discordance and eventually in percentage of concordance.
